# Dysbiosis of the Saliva Microbiome in Patients With Polycystic Ovary Syndrome

**DOI:** 10.3389/fcimb.2020.624504

**Published:** 2021-02-16

**Authors:** Na Li, Yangyang Li, Chen Qian, Qing Liu, Wei Cao, Mo Ma, Rongbo He, Rourou Chen, Rong Geng, Yu Liu

**Affiliations:** Department of Endocrinology, Sir Run Run Hospital, Nanjing Medical University, Nanjing, China

**Keywords:** polycystic ovary syndrome, salivary microbiome, fecal microbiota, 16S rRNA, diurnal rhythm

## Abstract

Significant differences in salivary microbiota communities between polycystic ovary syndrome (PCOS) patients and healthy controls have been reported, and interestingly, some salivary microbiota exhibit diurnal oscillation in healthy people. However, whether the diurnal oscillation of salivary microbiota is present in PCOS patients is unknown. In this study, we describe the differences in the saliva microbiome between the PCOS group and the control group at different time points over 24 h. 16S rRNA gene amplicon sequencing was performed on salivary and fecal samples from 10 PCOS patients and 10 healthy controls, and salivary samples were collected at 6-h intervals over 24 h (Zeitgeber (ZT)0, ZT6, ZT12, and ZT18). Among the salivary samples, those from the PCOS group showed significant differences from those of the control group at each time point. Differences were evident in taxa level and metabolic pathways. Interestingly, we found that PCOS disrupted the diurnal rhythm of the salivary microbiota abundance, as determined in the group of healthy women. In addition, no similar changes were found in PCOS patients and controls between the oral and fecal microbiota, including differential microbiota at the phylum level. In this study, significant differences in the composition of the salivary microbiota between PCOS and healthy women were detected at different time points. We also showed that the diurnal rhythm of relative abundance of the salivary microbiota was disrupted in patients with PCOS, which might be related to development of oral-related diseases and systematic metabolic disorders.

## Introduction

Polycystic ovary syndrome (PCOS) is one of the most common endocrine and metabolic disorders characterized by hyperandrogenism, oligo- or anovulatory and insulin resistance (IR), affecting 6.5%–8% of reproductive-age women (Norman et al., 2007). At present, the etiology of PCOS remains unclear, although the pathogenesis has been suggested to be multifactorial, involving genetic and environmental factors (Dumesic et al., 2015). In addition, the abnormal pulse secretion of gonadotropin-releasing hormone and luteinizing hormone (LH) is considered to be an important pathophysiological mechanism of PCOS (Roland and Moenter, 2014), as well as obesity, IR, and genetic defects (Dumesic et al., 2015). Tremellen and colleagues (Tremellen and Pearce, 2012) proposed that dysbiosis of the gut microbiota contributes to the pathogenesis of PCOS. Oral flora and intestinal flora are the two main components of the microecosystem in the human body. Increasing evidence from a large number of studies indicates that significant differences exist in the composition of the intestinal microbiota between PCOS and healthy women (Lindheim et al., 2017; Liu et al., 2017; Torres et al., 2018) and *Bacteroides vulgatus* has been shown to cause PCOS-like manifestations, such as polycystic ovaries and disorders of the oestrus cycle, in a mouse model (Qi et al., 2019). However, differences in the composition of microbiotas were inconsistent among studies.

Recent studies have suggested that the oral cavity serves as a reservoir for potential intestinal pathobionts that can exacerbate intestinal disease or other inflammatory diseases (Atarashi et al., 2017). The salivary microbiota composition and diversity have also been linked to PCOS (Akcali et al., 2014; Lindheim et al., 2016) and other metabolic alterations (Takeshita et al., 2016). PCOS could quantitatively affect the composition of the salivary microbiota and the relative abundance of salivary *Actinobacteria* was reduced in PCOS patients compared with healthy controls (Lindheim et al., 2016). Interestingly, Takayasu and co-workers (Takayasu et al., 2017) demonstrated that the majority of the salivary microbiota exhibited circadian oscillation in relative abundance within 24 h in healthy people. It remains unclear whether circadian oscillation of salivary microbes in PCOS patients also exists.

In this study, we analyzed and compared the microbiological composition of saliva and fecal samples by 16S rRNA gene amplicon sequencing between PCOS patients and healthy controls, and investigated the circadian oscillation of salivary microbes in PCOS patients.

## Materials and Methods

### Study Subjects and Sample Collection

This study was approved by the Ethics Committee of Sir Run Run Hospital of Nanjing Medical University (Permit number: 2019-SR-025). In total, 10 women with PCOS were recruited from the inpatient clinic of the Department of Endocrinology, Sir Run Run Hospital, aged from 18 to 45, and these patients were consecutively included from July 2019 to December 2019. The inclusion criteria were PCOS patients who were diagnosed according to the Rotterdam Criteria, who met two out of three of the following criteria: clinical/biochemical hyperandrogenism, oligo-/anovulation, and polycystic ovaries (Rotterdam, 2004). Patients with the following conditions were excluded: Cushing’s syndrome, thyroid disorder, hyperprolactinemia, congenital adrenal hyperplasia, and androgen-secreting tumors, as determined by clinical examination and appropriate laboratory tests. We included 10 healthy control subjects who fulfilled the established criteria, such as no signs of hyperandrogenism and any reported menstrual irregularity. For all study participants, the exclusion criteria included the use of oral contraceptives, other steroid hormones, metformin, and any other treatment within 3 months. None of the subjects were pregnant or had any form of gastrointestinal or periodontal disease, dental caries, any active infection of any type or a body mass index (BMI) <18 kg/m^2^. Before collecting the samples, the participants were asked to fill out a diet questionnaire. Those who met some criteria, including a regular diet and no history of alcohol, smoking, a special diet, or use of antibiotics or probiotics within 3 months, were entered into this study. Then, the participants received comprehensive dietary education and a standard diet was asked for taken at least three days at home before inpatient. The standard diet was formulated by nutritionist from Sir Run Run Hospital of Nanjing Medical University according to the Dietary Guidelines for Chinese Residents. During hospitalization, participants were fed nutritious meals. Samples were collected after obtaining informed consent from each study subject.

Fresh unstimulated saliva samples were collected every 6 h at 0:00 (Zeitgeber (ZT)0), 6:00 (ZT6), 12:00 (ZT12), and 18:00 (ZT18) for several minutes each time, accounting for a total of four samples for each subject. The participants slept from 0:00 to 6:00. The participants were not allowed to brush their teeth, drink beverages, or eat food for 2 h before sampling. Saliva was sampled after placing the tongue on the roof of the mouth for several minutes and then it was sampled directly into sterile tubes until the desired volume of approximately 2 ml was reached. Fecal samples were collected using stool collection tubes and all of salivary and fecal samples were kept on ice before being stored at −80°C within 1 h of collection, for subsequent use in DNA preparation. The venous blood samples were collected in the morning after 12 h of overnight fasting on the third to fifth days of the menstrual cycle, or during amenorrhoea after excluding pregnancy. Serum was prepared by centrifugation (3000 rpm for 10 min, 4°C) within 1 h and was then used for later analysis.

### Laboratory Measurements and the Calculation of Terms

The concentrations of serum hormones [total testosterone, oestradiol, progesterone, luteinizing hormone (LH), follicle stimulating hormone (FSH), sex hormone-binding globulin (SHBG) and cortisol] were determined using chemiluminescence. Total cholesterol (TC), triglyceride (TG), high-density lipoprotein (HDL), and low-density lipoprotein (LDL) were measured by enzymatic assays. The fasting blood glucose (FBG) concentration was quantified using the glucose oxidase method and the fasting insulin (FINS) level was measured by a radioimmunoassay. Body mass index (BMI = weight (kg)/height (m^2^)) was calculated. The homeostatic model assessment (HOMA) for insulin resistance was calculated using the formula [insulin (mU/L) × glucose (mM/L)]/22.5.

### DNA Extraction and 16S rRNA Amplicon Sequencing Analysis

We performed 16S rRNA gene amplicon sequencing to assess the bacterial composition and bacterial taxa, including phylum, class, order, family, and genus. Total genomic DNA from samples was extracted following the CTAB/SDS method. DNA purity and concentrations were assessed by 1% agarose gel electrophoresis. The concentration of DNA was diluted to 1 ng/µl in sterile water and the 16S rRNA genes of the V4 region were amplified using specific primers with barcodes for each saliva sample. The PCR mixtures contained 15 µl of Phusion^®^ High-Fidelity PCR Master Mix (New England Biolabs, US), 10 ng of template DNA, and 0.2 µM of forward and reverse primer, according to the manufacturer’s protocol. Thermal cycling consisted of an initial denaturation step at 98°C for 1 min, followed by 30 cycles of denaturation at 98°C for 10 s, annealing at 50°C for 30 s, and elongation at 72°C for 30 s, with a final extension step of 72°C for 5 min. PCR products were mixed equally, electrophoresed on 2% agarose gels, and then purified using a Qiagen Gel Extraction Kit (Qiagen, Mannheim, Germany). Finally, a cDNA library was constructed using the TruSeq^®^ DNA PCR-Free Sample Preparation Kit and was then quantified using a Qubit@ 2.0 Fluorometer (Thermo Fisher Scientific) and the Agilent Bioanalyzer 2100 system. Finally, sequencing of the library was carried out using the Illumina NovaSeq platform.

The paired-end reads of each sample were split according to the barcode and primer sequences and then spliced to generate the raw tags using FLASH (v1.2.7) software (Bokulich et al., 2013). After filtering the raw tag data, high quality clean data were obtained for subsequent analysis according to the QIIME v1.9.1 quality controlled process (Caporaso et al., 2010). The raw tags were compared with the Silva database *via* the UCHIME algorithm (Rognes et al., 2016) to detect chimeric sequences. Then, chimeras were removed and effective tags were finally obtained. Sequence analysis was carried out using Uparse v7.0.1001 software (Edgar, 2013) into operational taxonomic units (OTUs) with 97% similarity. Representative sequences for each OTU were screened for further annotation using the Silva database (Quast et al., 2013), which was based on the Mothur algorithm. OTU abundance information for each sample was then normalized using the least sequences of the sample as a standard and subsequent analysis of alpha and beta diversity was performed according to the output normalized data. Alpha diversity was calculated using QIIME v1.7.0 to analyze the complexity of species diversity for each sample using the Shannon and Chao1 indexes, and the results were displayed using the R software v2.15.3. Beta diversity analysis was performed to evaluate differences in species complexity between samples. Qiime software v1.9.1 was used to calculate the UniFrac distances and construct a phylogenetic tree for the UPGMA samples.

Linear discriminant analysis effect size (LEfSe) analysis was performed as previously reported to determine the significantly important microbial taxa (Segata et al., 2011). To explore the functional profile of the bacterial community data set, we applied a bioinformatic tool to predict the gene family abundance on the basis of a 16S gene survey and the database of phylogenetically referenced genomes (Phylogenetic Investigation of Communities by Reconstruction of Unobserved States, PICRUSt) (Langille et al., 2013).

### Statistical Analysis

Parameters or non-parameters in the demographics and clinical examination of all subjects were analyzed by the Student’s *t* test or Mann-Whitney U test using SPSS software v.22 and statistical significance was considered at *P* < 0.05. For the diurnal rhythm of some salivary bacteria, further analysis showed that data could not be obviously fit to a cosine function, which could be related to the number of time points and the limited sample size in this study. Similarly, the limited sample size and number of time points reduced the utility of using JTK/MetaCycle (Wu et al., 2016) for analysis. Therefore, SPSS software v.22 was used for repeated measures (RM) 1-factor ANOVA; the Friedman test; and Tukey, Dunn, and Bonferroni multiple comparison *post hoc* tests. The relationships between hormones and the diurnal rhythm of some salivary bacteria were analyzed by Pearson correlation tests.

## Results

### Characteristics of the Study Subjects

We collected a total of 80 unstimulated salivary samples (four samples per subject) every 6 h for one day from 10 healthy controls and 10 PCOS patients. The clinical data are summarized in **Table 1**. Overall, there was no significant difference in age or BMI between the two groups. Patients with PCOS had significantly higher total testosterone levels, and LH and LH/FSH ratios (*P* = 0.02, 0.02, and <0.001, respectively), whereas no difference was found for FBG, FINS, and HOMA-IR (*P* = 0.58, 0.24, and 0.20, respectively). In addition, no significant differences were detected in TC, TG, HDL, or LDL levels between the two groups (*P* = 0.55, 0.14, 0.32, and 0.19, respectively).

**Table 1:** Clinical characteristics of the PCOS and control groups.

Clinical measurements	Control (n = 10)	PCOS (n = 10)	*p*-value
Age (years)	31.5 ± 5.6	29.4 ± 5.9	0.42
BMI (kg/m^2^)	24.65 ± 3.23	26.89 ± 5.59	0.29
Testosterone (ng/ml)	1.26 ± 0.56	2.92 ± 1.75	0.02*
Oestradiol (pg/ml)	156.01 ± 32.56	188.70 ± 88.29	0.66
Progesterone (ng/ml)	1.44 ± 0.51	5.72 ± 8.65	0.78
LH (IU/L)	3.30 ± 1.12	12.86 ± 9.43	0.02*
FSH (IU/L)	6.42 ± 1.19	4.99 ± 2.51	0.16
LH/FSH	0.53 ± 0.17	2.44 ± 1.22	0.00*
SHBG (nmol/L)	44.20 ± 20.73	30.98 ± 18.45	0.11
Cortisol (nmol/L)(ZT0)	62.87 ± 29.06	60.53 ± 30.34	0.74
Cortisol (nmol/L)(ZT6)	309.25 ± 86.47	265.62 ± 125.41	0.32
Cortisol (nmol/L)(ZT18)	138.34 ± 46.75	110.51 ± 47.61	0.22
TC (mmol/L)	4.20 ± 1.05	4.48 ± 0.80	0.55
TG (mmol/L)	0.96 ± 0.38	1.43 ± 0.77	0.14
HDL (mmol/L)	1.42 ± 0.24	1.24 ± 0.43	0.32
LDL (mmol/L)	2.47 ± 0.87	3.00 ± 0.75	0.19
FBG (mmol/L)	4.87 ± 0.81	4.98 ± 0.79	0.58
FINS (uIU/ml)	8.65 ± 4.03	12.47 ± 8.39	0.24
HOMA-IR	1.83 ± 0.87	2.86 ± 2.13	0.20

Student’s t-test for normal distributions, Mann-Whitney U test for non-normal distributions, mean ± standard deviation; *P < 0.05 was considered statistically significant.

PCOS, polycystic ovary syndrome; Control, healthy people; Clinical measurements, Clinical parameters.

## Analysis of the Bacterial Counts in Saliva in the PCOS and Control Groups at Different Time Points

### Alpha Diversity of the Salivary Microbiome in Each Group at Different Time Points

An average of 69,083 tags was obtained from each sample. An average of 66,565 effective data were obtained after quality control and these reads were clustered into 3,175 OTUs with a 97% identity of coverage for each group.

Salivary samples were obtained every 6 h over a 24-h period from individual healthy controls and PCOS patients. The healthy controls or PCOS patients at different time points shared a large number of OTUs (609 and 454 OTUs, respectively), which are represented by the overlapping areas of the circles in **Supplemental Figures 1A, B**. The different numbers of OTUs in the Control.ZT0 vs. PCOS.ZT0, Control.ZT6 vs. PCOS.ZT6, Control.ZT12 vs. PCOS.ZT12, and Control.ZT18 vs. PCOS.ZT18 are exhibited in **Supplemental Figures 1C–F**, in which significant differences in OTUs between PCOS patients and controls are evident. Moreover, the rarefaction curve for the number of observed species per sample was close to a plateau over 10,000 to 40,000 sequence reads (**Supplemental Figure 2**).

The salivary bacterial community in the controls and PCOS patients at different time points was then analyzed quantitatively using the Shannon and Chao1 indexes, respectively. The patients in the PCOS group had lower alpha diversity than those in the control group at ZT0 (*P* < 0.05, **Table 2**). However, at other time points, no differences in the alpha diversity parameters were observed between PCOS patients and the controls (**Table 2**). The results revealed that microbial diversity was dependent on the time point of sampling during the day.

**Table 2:** Alpha diversity differences in the salivary microbiota between the PCOS and control groups at each time point.

Time of day	Alpha diversity index	Control	PCOS	*p*-value
ZT0	Shannon	4.93 ± 0.51	4.04 ± 0.84	0.01*
	Chao1	574.90 ± 366.15	325.09 ± 88.28	0.02*
ZT6	Shannon	4.74 ± 0.50	4.63 ± 0.72	0.69
	Chao1	464.70 ± 221.84	350.59 ± 62.50	0.12
ZT12	Shannon	4.79 ± 0.71	4.79 ± 049	0.71
	Chao1	506.91 ± 148.68	429.98 ± 116.51	0.39
ZT18	Shannon	4.78 ± 0.50	4.57 ± 0.74	0.49
	Chao1	456.17 ± 135.74	360.30 ± 137.01	0.13

In total, 40 saliva samples were examined per analysis. The number of saliva samples per time point per group = 10. Student’s t test for normal distributions, Mann-Whitney U test for non-normal distributions, mean ± standard deviation; *P < 0.05 was considered statistically significant. Time of day, Time point of day (ZT0, 0:00; ZT6, 6:00; ZT12, 12:00; ZT18, 18:00); PCOS, polycystic ovary syndrome; Control, healthy people；Alpha diversity index, Shannon index reflects species diversity and Chao1 index reflects species richness.

### Relative Abundance of Microbiota in Each Group at Different Time Points

**Figure 1:**
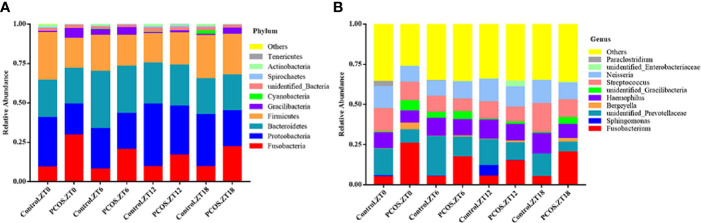
Relative abundance of salivary microbiota at different taxomal levels and comparison between the control and PCOS groups at different time points. The mean relative abundances at the phylum **(A)** and genus **(B)** levels in different groups are represented. Different colors represent different microbiota, and area sizes represent the relative content of the microbiota composition.

To further explore the distribution and divergence of the microbial community in the saliva of each group, we summarized the relative abundance of the microbiota at different taxonomic levels. The PCOS and control groups showed similar patterns of dominant bacteria at the different time points, but significant differences between the groups were evident at phylum and genus levels for each time point (**Figure 1**). At the phylum level, for the PCOS.ZT0, PCOS.ZT6, PCOS.ZT12, and PCOS.ZT18 groups, the relative abundances of Fusobacteria were increased compared with those in the Control.ZT0, Control.ZT6, Control.ZT12, and Control.ZT18 groups, respectively (**Figure 1A**). Meanwhile, at the genus level, the relative abundances of Fusobacterium in the PCOS groups were higher than in the control groups (**Figure 1B**).

### Beta Diversity of the Salivary Microbiome Between the Control and PCOS Groups at Different Time Points

**Figure 2:**
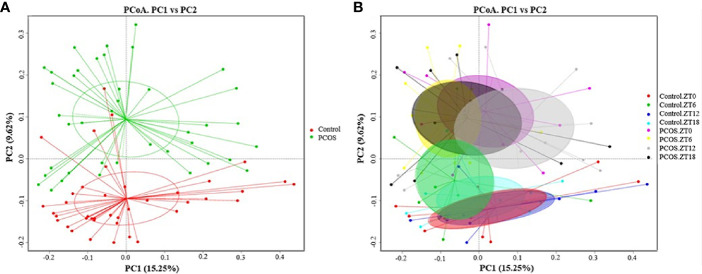
Differences in the diurnal oral microbe community structure between the control group and the PCOS group. PCoA of the salivary pellets colored by different groups **(A)** collected over 24 h from healthy controls and PCOS patients, analyzed using the unweighted UniFrac method. **(B)** PCoA analysis of the salivary microbiota, colors represent Zeitgeber (ZT) times.

Since the major purpose of our study was to investigate differences in the salivary microbiome of PCOS patients compared with healthy controls, we focused on investigating the OTU-based beta diversity of the microbial community in PCOS patients’ saliva and compared it with that of the controls. Assessment of the 16S rRNA V3–V4 region *via* Illumina MiSeq sequencing showed significant differences between the PCOS and control groups in microbial community composition, as shown by PCoA of the abundance-unweighted beta diversity (**Figure 2A**). Further assessment of beta diversity in the salivary microbiome between the PCOS and control groups showed temporal shifts over 24 h that were more evident in the control group (**Figure 2B**). When the beta diversity of each group was analyzed using the phylogenetic weighted UniFrac-based method, significant differences were only observed when comparing groups Control.ZT0 and PCOS.ZT0, and Control.ZT18 and PCOS.ZT18 (*P* < 0.05, **Table 3**). However, no significant differences between these groups were detected when using the phylogenetic unweighted UniFrac-based method (**Table 3**). To investigate the similarities between different samples, we used weighted and unweighted UniFrac distance matrixes for UPGMA cluster analysis, which revealed two distinct clusters representing the PCOS and control groups (**Supplemental Figure 3**). Significantly different compositions of the salivary microbiota were detected for the two groups at different time points, suggesting that different time points could influence the composition of the salivary microbiota.

**Table 3:** Comparison of beta diversity between the PCOS and control groups using unweighted and weighted UniFrac distances.

Group	Unwei-unifrac	Wei-unifrac
Control.ZT0 vs. PCOS.ZT0	0.91	0.03*
Control.ZT6 vs. PCOS.ZT6	0.93	0.41
Control.ZT12 vs. PCOS.ZT12	0.58	0.98
Control.ZT18 vs. PCOS.ZT18	0.11	0.047*

In total, 40 saliva samples were examined per analysis. Number of saliva samples per time point per group = 10. Student’s t test for normal distributions, Mann-Whitney U test for non-normal distributions, mean ± standard deviation; *P < 0.05 was considered statistically significant. Group, PCOS group and control group were grouped according to time points of day; Unwei-unifrac and Wei-unifrac represent beta diversity.

### Differential Microbial Composition in Each Group at Different Time Points

**Figure 3:**
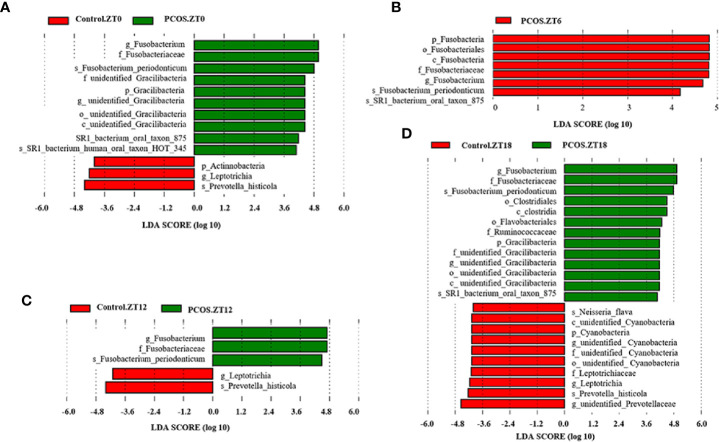
Identification of specific bacterial taxa in PCOS patients and controls at different time points. Linear discriminant analysis (LDA) effect size (LEfSe) was used to identify biomarkers with significant differences between the two groups: Control.ZT0 vs. PCOS.ZT0 **(A)**, Control.ZT6 vs. PCOS.ZT6 **(B)**, Control.ZT12 vs. PCOS.ZT12 **(C)**, and Control.ZT18 vs. PCOS.ZT18 **(D)**. LDA score assessed the size of the differentiation between the two groups with a score threshold of 4.0. The enriched taxa in the PCOS group (right side) are shown with a positive LDA score, whereas the taxa increased in the control group (left side) have a negative score.

To identify the specific bacterial taxa present under different conditions at different time points, the composition of the microbiota was compared for each group by an LEfSe assay and LDA score assessments of the size of the differentiation between two groups with a score threshold of 4.0. As shown in **Figure 3**, with the exception of Fusobacterium, there were some significant differences between the PCOS and control groups at different time points. At the ZT0 time point, the relative abundances of *Gracillibateria* (including the phylum, class, order, family, and genus) in PCOS patients were higher than those in the healthy controls, but the relative abundances of p_*Actinobacteria* and g_*Leptotrichia* in PCOS patients were lower than in the controls (**Figure 3A**). At the ZT6 time point, the relative abundances of p_*Fusobacteria*, o_*Fusobacteriales*, and c_*Fusobaterila* in the PCOS group were higher than those in the control group (**Figure 3B**). At the ZT12 time point, the relative abundance of g_*Leptotrichia* in PCOS patients was lower than in the controls (**Figure 3C**). Interestingly, there was large divergence in the microbial community at the ZT18 time point. A higher relative abundance of o_*Clostridiales*, c_*Clostridia*, o_*Flavobacteriales*, f_*Ruminococcaceae*, and *Gracilibacteria* in the PCOS group was found. However, the relative abundance of p_*Cyanobacteria*, f_*Leptotrichiaceae*, g_*Leptotrichia*, and g_unidentified-*Prevotellaceae* in the PCOS group was lower than that in the control group (**Figure 3D**). Taken together, our data revealed that the PCOS and control subjects could be distinguished at different time points based on their oral microbiota.

## Analysis of the KEGG Metabolomic Pathway in the PCOS and Control Groups at Different Time Points

**Figure 4:**
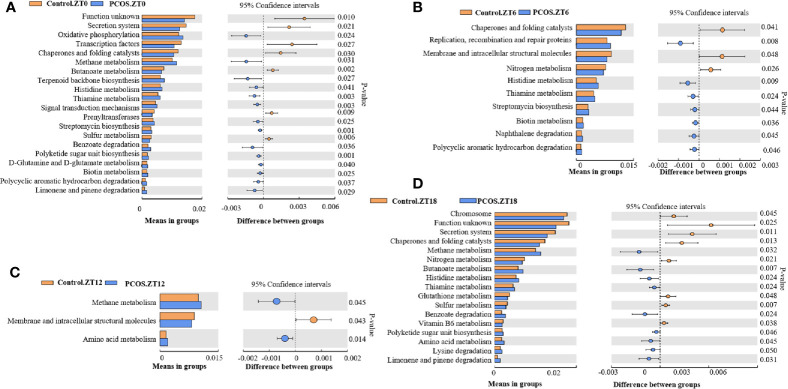
KEGG pathway analysis showing significant differences between the PCOS and control groups at different time points. Functional prediction of the altered salivary microbiota between the two groups by PICRUSt analysis was based on KEGG pathways: Control.ZT0 vs. PCOS.ZT0 **(A)**, Control.ZT6 vs. PCOS.ZT6 **(B)**, Control.ZT12 vs. PCOS.ZT12 **(C)**, and Control.ZT18 vs. PCOS.ZT18 **(D)**. Differences were considered significant at *p* < 0.05 using the Student’s t-test.

PICRUSt analysis was used to explore changes in the KEGG pathway induced by the altered composition of salivary bacteria. Compared with the control group at the ZT0 time point, multiple pathways appeared to be affected in the PCOS group. Three pathways involving “secretion system”, “transcription factors”, and “chaperones and folding catalysts” were downregulated in the PCOS group, and two pathways including “oxidative phosphorylation” and “methane metabolism” were markedly upregulated (**Figure 4A**). At the ZT6 time point, PCOS led to the upregulation of the pathway “replication, recombination and proteins” and the downregulation of three metabolic pathways, including “chaperones and folding catalysts”, “membrane and intracellular structural molecules”, and “nitrogen metabolism” (**Figure 4B**). At the ZT12 time point, “methane metabolism” and “amino acid metabolism” were upregulated in the PCOS group compared with the control group, and “membrane and intracellular structural molecules” was downregulated (**Figure 4C**). Finally, at the ZT18 time point, we found that some pathways including “chromosome”, “secretion system”, “chaperones and folding catalysts”, and “nitrogen metabolism” were markedly downregulated, while “methane metabolism” and “butanoate metabolism” were upregulated in the PCOS group (**Figure 4D**). Taken together, these data revealed that “methane metabolism” was upregulated consistently at several time points (except for ZT6), and “chaperones and folding catalysts” was downregulated consistently (except for ZT12) in the PCOS group. In addition, three metabolic pathways including “secretion system”, “membrane and intracellular structural molecules”, and “nitrogen metabolism” were markedly downregulated at ZT0 and ZT18, ZT6 and ZT12, and ZT6 and ZT18, respectively.

In summary, significant differences were evident between the PCOS and control groups for each time point at the different taxa levels and for different metabolic pathways, suggesting that the abundance of some salivary microbiota could be circadian.Trish

### Rhythm of Salivary Microbiota Between the PCOS and Control Groups at Different Time Points

**Figure 5:**
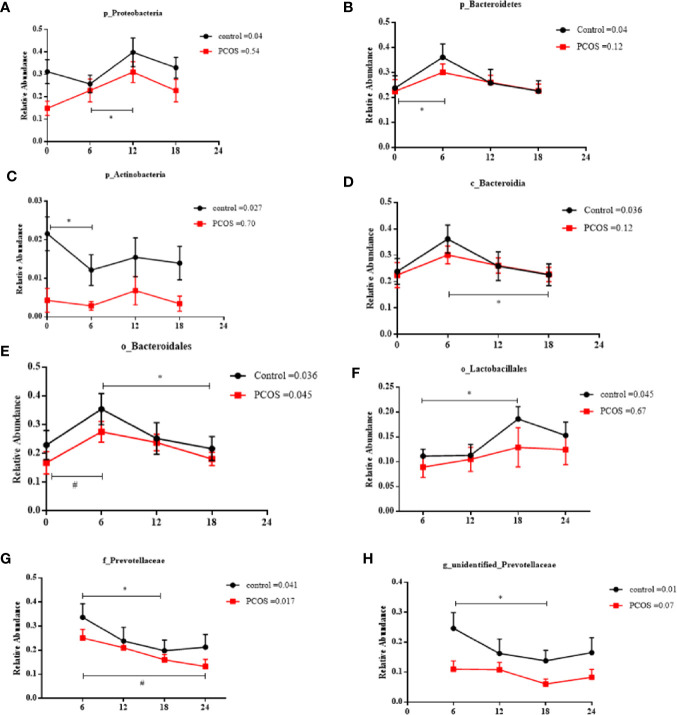
Relative abundance of significant taxa in the PCOS and control groups across the time points. The diurnal oscillation of salivary microbiota was present in the control and PCOS groups, including p_*Proteobacteria*, p_*Bacteroidetes*, p_*Actinobacteria*, c_*Bacteroidales*, o_*Bacteroidales*, o_*Lactobacillales*, f_*Prevotellaceae* and g_unidentified-*Prevotellaceae*. The RM Friedman test indicated that various individual taxa were significantly different across time points in the control group **(A–H)** and the PCOS group **(E**, **G)**. * or ^#^ Dunn’s multiple comparison test indicated significant differences between time points in either the control or PCOS group (* represents the control group and ^#^ represents the PCOS group). Error bars=SEM.

Next, we assessed the diurnal rhythm of the salivary microbiota. The time of day was a significant factor in the relative abundances of some individual bacteria at the taxonomic levels of phylum, class, order, family, and genus. Specifically, the following taxa exhibited a significant diurnal rhythm in the control group (**Figures 5A–H**): p_*Proteobacteria*, p_*Bacteroidetes*, p_*Acidobacteria*, c_*Bacteroidales*, o_*Bacteroidales*, o_*Lactobacillales*, f_*Prevotellaceae*, and g_unidentified-*Prevotellaceae*; and in the PCOS group (**Figures 5E, G** **)**: o_*Bacteroidales* and f_*Prevotellaceae*. The relative abundances of some bacteria were not diurnal in the control group or the PCOS group (**Supplemental Figure 4**), such as p_*Fusobacteria*, g_*Fusobacterium*, and p_*Gracilibacteria*. These results showed that the diurnal rhythm observed for some bacterial taxa in the salivary microbiota of healthy controls, was not present in the PCOS patients.

PCOS is characterized by endocrine disorders. To further investigate whether the diurnal rhythm of the salivary microbiota was related to hormone levels, we performed a Pearson correlation test. No significant correlation between hormone levels (including cortisol, LH, FSH, oestradiol, testosterone, progesterone, and FINS) and the diurnal rhythm of the salivary microbiota was detected (**Supplemental Table 1**).

### Comparing the Composition of the Microbiota Between Saliva and Feces in the PCOS and Control Groups

To investigate the correlation between the salivary and fecal microbiotas, we collected fecal samples from the participants. No significant effect of PCOS was observed for bacterial alpha diversity (**Supplemental Figure 5A**) or beta diversity (**Supplemental Figure 5B**). At the phylum level, the relative abundance of Firmicutes was reduced and the relative abundance of *Bacteroidetes* was increased in PCOS patients compared with the controls using the fecal samples (**Supplemental Figure 5C**), whereas in the salivary samples, the relative abundance of *Fusobacteria* was increased in PCOS patients compared with the controls (**Supplemental Figure 5D**). LEfSe was used to identify significant differences in the microbiota between the two groups using the fecal samples, and g_*Blautia*, g_*Streptococcus*, and f_*Streptococcaceae* were less abundant in the PCOS group than in the control group (**Supplemental Figure 5E**), indicating that there were no common differences between the fecal and oral microbiotas of the two groups (**Figure 3**).

## Discussion

Recently, several reports have shown that the composition of the intestinal and salivary microbiotas in PCOS patients was significantly different from that of healthy people (Akcali et al., 2014; Lindheim et al., 2016; Lindheim et al., 2017). The microbiotas have also been reported to exhibit diurnal oscillation in healthy people and mice (Leone et al., 2015; Takayasu et al., 2017). In this study, we investigated differences in the microbial community of saliva between PCOS patients and controls at different time points, and then explored the differences between the salivary and fecal microbiotas between the two groups. We found that the salivary microbiota from the PCOS group had lower alpha diversity than that of the control group at ZT0 only, and significant differences in beta diversity were observed in the salivary samples between PCOS patients and controls at the ZT0 and ZT18 time points, respectively. In addition, significant differences were detected between the PCOS and control groups at the taxa level and among metabolic pathways at each time point for the salivary samples, but the relative abundance of *Fusobacterium* was increased in PCOS patients at each time point. Interestingly, we showed for the first time that PCOS could disrupt the diurnal rhythm of the salivary microbiota, which may be one of the reasons for the differences in bacterial abundance between the two groups at different time points.

Many factors affect the composition of the oral microbiota, including endogenous and exogenous factors. In this study, the following variables were ruled out due to sampling: diet, sleep, antibiotics, smoking, drinking, and some oral diseases. In a previous report, Lindheim and colleagues (Lindheim et al., 2016) suggested that there was no significant difference between PCOS patients and controls regarding alpha and beta diversity of the salivary microbiota in the morning, which was consistent with the results of this study at the ZT6 time point. However, PCOS patients showed lower alpha diversity than the control group at ZT0 and significant differences in beta diversity were observed at ZT0 and ZT18 in this study. This may be due to the effects of the clock on the composition of the microbiota, suggesting that we should select an appropriate time point for sampling. We showed that the relative abundance of *Fusobacterium* in the PCOS groups was higher than in the control groups at each time point. *Fusobacterium*, a phylum of Gram-negative bacteria, can increase the risk of some diseases, such as periodontitis (Kamma et al., 1995), local skin diseases (Kanoe et al., 1995), and obesity (Genco et al., 2005). In addition, a previous study revealed that PCOS was associated with a decreased relative abundance of salivary *Actinobacteria* (Lindheim et al., 2016). Consistent with this article, we also found that the relative abundance of *Actinobacteria* was lower in the PCOS group compared with the control group at the ZT0 time point. *Actinobacteria* is a common constituent of the microbiotas of the oral cavity and skin, and was reported to be reduced during periodontal disease (Liu et al., 2012; Wang et al., 2013). As our PCOS patients were periodontally healthy, we hypothesized that the increase in the relative abundance of *Fusobacterium* and the reduction in the relative abundance of *Actinobacteria* may disrupt the oral environment and eventually result in periodontal disease. This is consistent with the higher reported prevalence of periodontal disease in PCOS women compared with healthy controls (Porwal et al., 2014). In addition, functional prediction showed that carbohydrate-related metabolism, such as “methane metabolism”, was significantly higher in the PCOS group at certain time points, and “methane production” has been shown to be associated with intestinal diseases, including inflammatory bowel diseases and colon cancer (Roccarina et al., 2010). Whereas, at some time points, “chaperones and folding catalysts”, “secretion system”, and “membrane and intracellular structural molecules” were markedly downregulated, which could influence the synthesis of proteins contributing to some metabolic diseases, such as obesity, IR, and fatty liver (Cao and Kaufman, 2013). Therefore, we speculated that the changes in the composition of salivary bacteria could aggravate metabolic disorders, potentially contributing to the development of PCOS. However, in this study, microbial function was estimated with PICRUSt software using phylogenetic affiliation of OTUs and a reference genome database. This assessment is limited to previously annotated genes (ignoring undiscovered functional genes), and does not account for potential differences in gene expression. Therefore, we hope to perform metagenomics and metatranscriptomics to obtain more precise results in the future.

Recent studies in animals have reported that the fecal microbiota exhibits a circadian rhythm, driven by the clock genes (Thaiss et al., 2014; Liang et al., 2015). Furthermore, 15% of the salivary microbiota [such as *Bacteroidetes* phyla (Takayasu et al., 2017)] has been reported to exhibit diurnal oscillation in healthy people (Dallmann et al., 2012). Therefore, the significant differences in taxa levels and metabolic pathways between PCOS patients and the controls at each time point may result from the diurnal rhythm of abundance of some salivary microbiota.

In the present study, we found that some salivary microbiota (p_*Proteobacteria*, p_*Bacteroidetes*, p_ *Actinobacteria*, c_*Bacteroidales*, o_*Lactobacillales*, and g_unidentified-*Prevotellaceae*) in the control group were rhythmic in their abundance, whereas this rhythm disappeared in PCOS patients. *Proteobacteria* have been reported to be associated with inflammation and immunity (Shin et al., 2015), whereas Bacteroidetes appeared to be enriched in patients suffering from irritable bowel syndrome (Pittayanon et al., 2019), and is involved in type 1 and type 2 diabetes, and obesity (Rajilic-Stojanovic and de Vos, 2014). *Lactobacillales* is considered a beneficial gut bacteria, playing a role in infection control, metabolism regulation, and inflammation/allergy modulation (Pessione, 2012). As mentioned above, *Actinobacteria*, which may be modulated by light, are able to coordinate organic carbon uptake and utilization, accompanied by the production of photosynthate that enhances growth during the daytime (Maresca et al., 2019). *Actinobacteria* have been found to be reduced during periodontal diseases (Kornman and Loesche, 1982; Kanoe et al., 1995) and metabolic diseases (Long et al., 2017). Disorders of diurnal oscillation in the salivary microbiota might be related to development of periodontal disease and some metabolic disorders in patients with PCOS. The effect of gonadal hormones on the microbiota has been reported since the 1980s (Kornman and Loesche, 1982). Certain bacterial phyla in saliva, such as *Bacteroidetes*, *Acidobacteria*, and *Proteobacteria*, have been positively correlated with oestradiol (Sohail et al., 2019) and exposure to cortisol in the oral microbiome can cause a significant shift in the gene expression profile of the community (Duran-Pinedo et al., 2018). Cortisol has a marked circadian rhythm and we investigated whether the diurnal rhythm of the abundance of the salivary microbiota was related to the circadian rhythm of cortisol. However, in this study, we found no significant correlation between hormones and the relative abundance of salivary microbiota. Therefore, we speculate that microbiota may have its own diurnal rhythm, which remain to be further investigated. Further insights may help PCOS patients to maintain oral health and prevent metabolic disorders.

Recent studies have shown that individuals with PCOS have gut microbiota communities that are different from those of healthy controls (Liu et al., 2017; Torres et al., 2018; Qi et al., 2019), but the differences in composition of microbiotas were inconsistent among studies. Some studies have suggested that the oral cavity serves as a reservoir for potential intestinal pathobionts that can exacerbate intestinal disease or other inflammatory diseases (Atarashi et al., 2017). To further investigate the relationship between the fecal and salivary microbiomes, we collected fecal samples from the participants to perform 16S rRNA gene amplicon sequencing. No differences in alpha or beta diversity were detected between PCOS patients and healthy controls. These results were in agreement with those of Insenser and co-workers (Insenser et al., 2018), but inconsistent with some other studies (Liu et al., 2017; Torres et al., 2018) that reported reductions in the alpha and beta diversity indexes in PCOS patients compared with controls. The fact that we did not find such a reduction might be related to the characteristics of the populations because the aforementioned studies did not include obese women, or might be related to differences in race, sample size, or other interferences. Importantly, we found other changes between the oral and fecal microbiotas of PCOS patients and controls, including phylum level differences, suggesting that location may have an impact on microbiota composition.

For a human population study on the diurnal rhythm of the microbiota, sampling of saliva is more convenient than feces, and we found significant differences between the salivary and fecal microbiotas. In addition, in this study, we detected differential microbiota at different time points and the diurnal rhythm of some microbiota in PCOS patients. Future studies may explore the effect of the clock on the mechanisms of PCOS, which may lead to new strategies for the prevention of metabolic disorders in PCOS patients.

Approximately 70% PCOS patients have been reported to have dyslipidemia and triglycerides is a main determinant of free testosterone index in PCOS (Hestiantoro et al., 2019). In addition, abnormal glucose tolerance and insulin resistance were observed in many PCOS patients (Bhathena, 2011; Jones et al., 2012). Recent study has revealed that the microbiota is subjected to variations in the host’s high triglycerides, fasting glucose and fasting insulin (Bhute et al., 2017), and metabolic disorders such as obesity (Turnbaugh et al., 2006) and diabetes (Qin et al., 2012; Karlsson et al., 2013) are found to be associated with microbial composition in which certain OTUs or species are present in different proportions. For example, some studies have suggested that higher abundance of phylum Firmicutes and lower abundance of phylum Bacteroidetes were associated with obesity (Backhed et al., 2004; Turnbaugh et al., 2006; Turnbaugh et al., 2009). In addition, a positive correlation was observed between Lactobacillus abundance and blood glucose levels (Karlsson et al., 2013), and the proportion of the phylum Firmicutes and the class Clostridia in the gut of diabetes patients was significantly reduced (Qin et al., 2012).A limitation of our study is the small sample size, which precluded stratification of PCOS subtypes by BMI or metabolic syndrome. In this study, BMI, age, and several metabolic factors of the two groups were matched to minimize the possible influence of these factors on the composition of the microbiota as much as possible. A large-scale study is required to confirm the preliminary interesting findings in this study. In addition, some oral health parameters (e.g., the plaque index and the simplified oral hygiene index), which could be related to composition of the microbiota as reported in some published studies (Takeshita et al., 2016; Frid et al., 2020) were not recorded. A more detailed oral examination would be helpful in the future. Furthermore, although the participants received comprehensive dietary education before being included in the study, the different eating behaviors of the subjects could have affected bacterial properties. In addition, we included only four saliva sampling time points (ZT0, ZT6, ZT12, and ZT18). Future studies should include more time points to better represent the diurnal rhythm, and allow the data to fit a cosine function or the JTK/MetaCycle.

Our findings revealed significant differences in the composition of the salivary microbiota between PCOS and healthy women, and we showed for the first time that the diurnal rhythm of some salivary bacteria was disrupted in PCOS patients, potentially leading to oral and metabolic disorders in PCOS patients. The impact of disruption of the daily rhythm of the salivary microbiota on the host’s metabolism should be explored in future studies and may aid new approaches to prevent oral-related diseases and systematic metabolic disorders in PCOS patients.

## Data Availability Statement

The data sets presented in this study can be found in online repositories. The names of the repository/repositories and accession number(s) can be found below: http://www.ncbi.nlm.nih.gov/bioproject/674750.

## Ethics Statement

The studies involving human participants were reviewed and approved by Sir Run Run Hospital, Nanjing Medical University. The patients/participants provided their written informed consent to participate in this study.

## Author Contributions

NL was involved in the analysis and interpretation of data and manuscript drafting. YL conceived the original idea and supervised the project. YL and YYL interpreted the data and critical revision of article. CQ, QL, WC, MM, RH, RC, and RG assisted in data collection. All authors contributed to the article and approved the submitted version.

## Funding

This study was supported by the National Key R&D Program of China (2016YFC1305000, 2016YFC1305005), Innovative and entrepreneurial team of Jiangsu Province (2018), National Natural Science Foundation of China (81770778), the Science and Technology Plan of Jiangsu Province-Social Development (BE2017738), and the Key Medical Talents Project of Jiangsu Province (ZDRCA2016088).

## Conflict of Interest

The authors declare that the research was conducted in the absence of any commercial or financial relationships that could be construed as a potential conflict of interest.
