# Supplementary material for: Dysbiosis of the Saliva Microbiome in Patients With Polycystic Ovary Syndrome
Source: Front Cell Infect Microbiol. 2021 Feb 16;10:624504. doi: 10.3389/fcimb.2020.624504 (PMC7921782; doi:10.3389/fcimb.2020.624504)
Supplement: Supplementary file 1 [file DataSheet_1.docx]

Supplementary Material

# Supplementary Figures and Tables

# Supplementary Figures


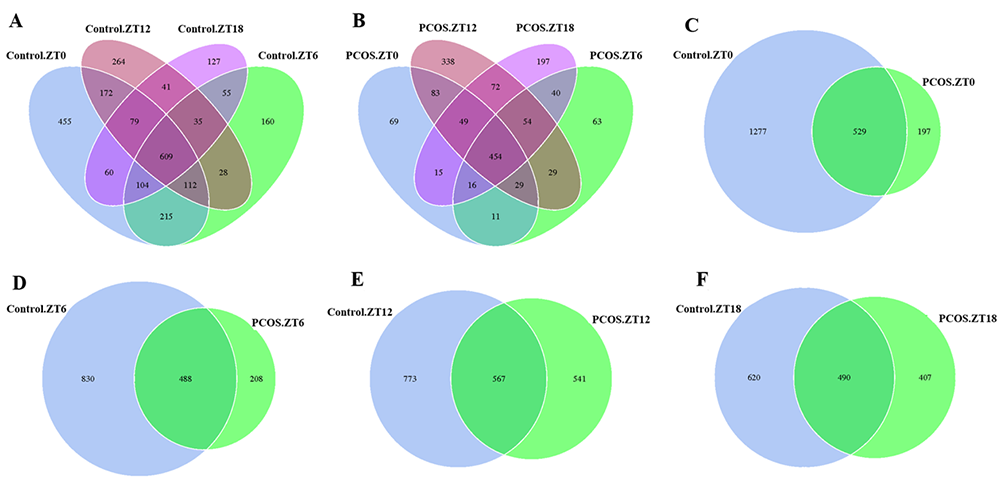


**Supplementary Figure 1.**  Distribution of observed salivary microbiota species in the PCOS and control groups at different time points. A Venn diagram illustrates observed overlaps of OTUs at a 97% similarity level for healthy individuals (A) or PCOS patients (B) at different time points, and for control and PCOS groups at ZT0 (C), ZT6(D), ZT12 (E), and ZT18 (F). The number of OTUs is indicated. The size of the circles is proportional to the number of OTUs.


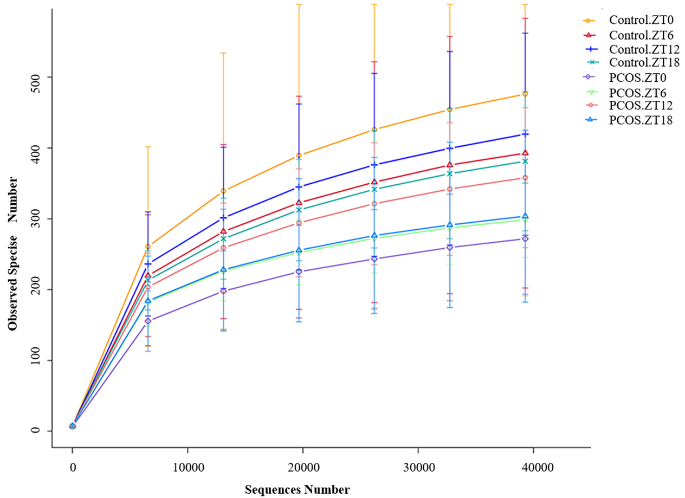


**Supplemental Figure 2.** Rarefaction curves for the number of observed OTUs per sample in the control and PCOS groups at different time points (ZT0, ZT6, ZT12, ZT18).


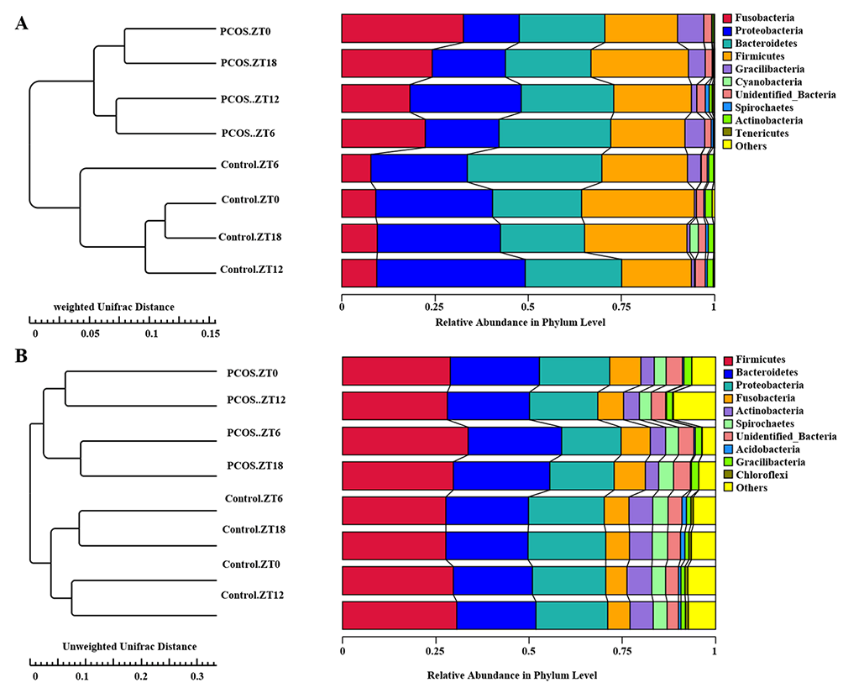


**Supplemental Figure 3.** UPGMA cluster analysis using a weighted UniFrac distance matrix (A) and an unweighted UniFrac distance matrix (B). The UPGMA clustering tree structure is shown on the left, and the relative abundance distribution of each sample at the phylum level is shown on the right.


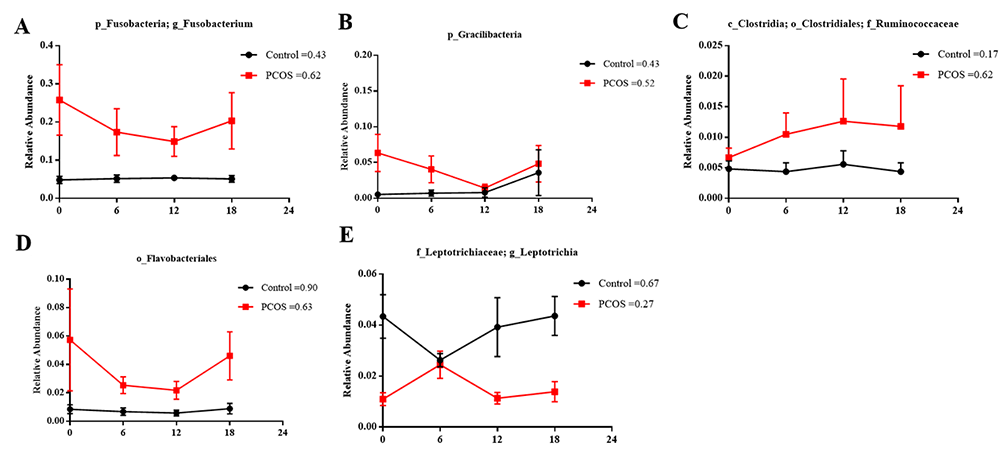


**Supplemental Figure 4.** Relative abundance of significant taxa in the control and PCOS groups across the time points. p_Fusobacteria;g_Fusobacterium (A), p_Gracilibacteria (B), c_Clostridia;o_Clostridiales;f_Ruminococcaceae (C), o_Flavobacteriales (D), and f_Leptotrichiaceae;g_Leptotrichia (E). The RM Friedman test indicated no differences in the various individual taxa across the time points in the control and PCOS groups. Error bars=SEM.


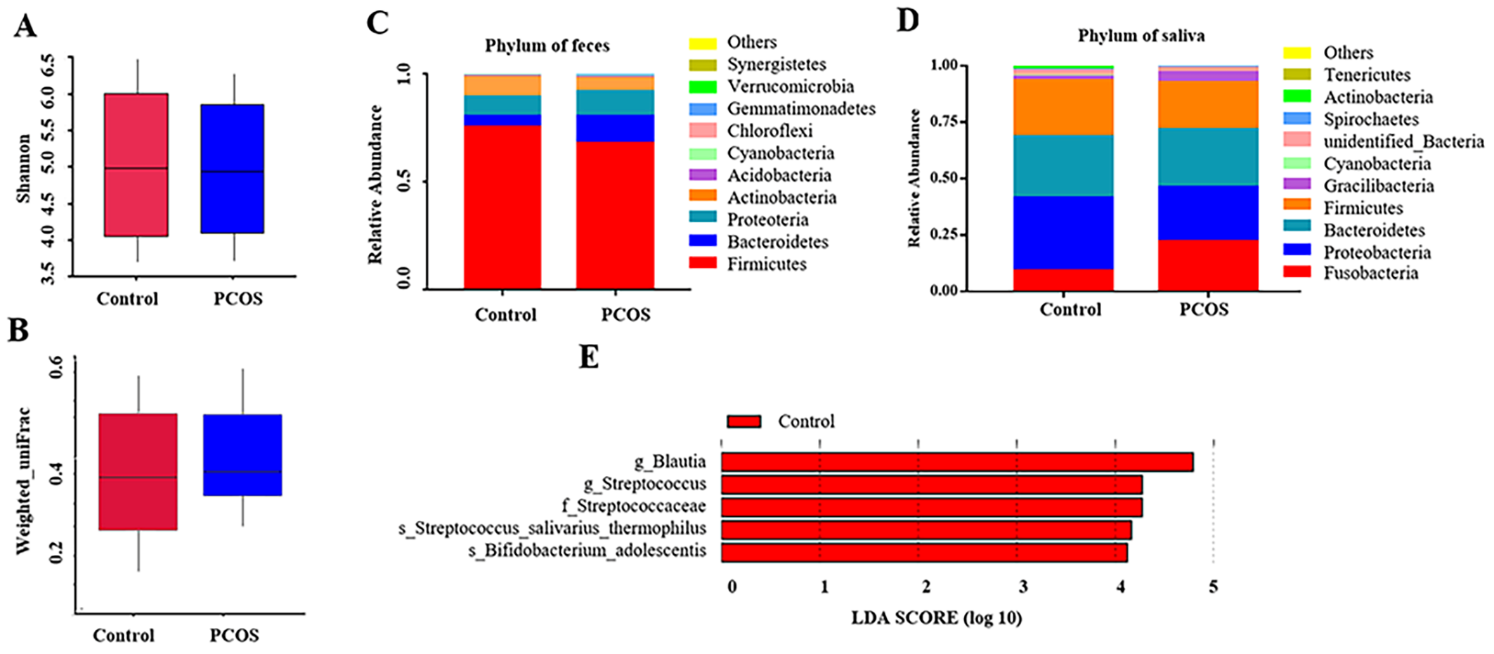


**Supplemental Figure 5.** Analysis of the 16S rRNA gene amplicon sequences derived from the faeces samples of PCOS patients and controls. (A) The alpha diversity of the faecal microbiota was evaluated by the Shannon index and analysed by an unpaired Student's t test (p >0.05). (B) Boxplot of the beta diversity in faecal samples based on the weighted UniFrac-based method (p >0.05). (C) The mean relative abundances at the phylum level of the faecal samples from the PCOS and control groups. (D) The mean relative abundances at the phylum level of the salivary samples from the PCOS and control groups. (E) The microbiota that showed significant differences between the two groups as analysed by LEfSe with an LDA score threshold of 4.0. The taxa enriched in the control group are shown.

**Supplemental Table 1.** **Correlations between hormones and the relative abundance of microbiota**

|  | | p_*Proteobacteria* | p_*Bacteroidetes* | p_*Acidobacteria* | | c_*Bacteroidia* | o_*Bacteroidales* | o_*Lactobacillales* | f_*Prevotellaceae* | g_unidentified_*Prevotellaceae* |
| --- | --- | --- | --- | --- | --- | --- | --- | --- | --- | --- |
| Cortisol  (ZT0) | r | -0.04 | 0.14 | 0.28 | 0.14 | | 0.35 | -0.03 | 0.42 | 0.4 |
|  | *p* | 0.87 | 0.6 | 0.27 | 0.6 | | 0.15 | 0.9 | 0.08 | 0.11 |
| Cortisol  (ZT6) | r | 0.24 | 0.19 | 0.31 | 0.19 | | 0.27 | -0.07 | 0.24 | 0.42 |
|  | *p* | 0.32 | 0.44 | 0.19 | 0.44 | | 0.27 | 0.77 | 0.31 | 0.07 |
| Cortisol  (ZT18) | r | -0.35 | 0.14 | -0.04 | -0.43 | | 0.27 | 0.30 | 0.29 | 0.29 |
|  | *p* | 0.16 | 0.58 | 0.87 | 0.07 | | 0.28 | 0.23 | 0.25 | 0.24 |
| LH | r | -0.42 | -0.08 | -0.34 | -0.08 | | -0.15 | -0.38 | -0.15 | -0.24 |
|  | *p* | 0.19 | 0.73 | 0.17 | 0.73 | | 0.55 | 0.10 | 0.53 | 0.32 |
| FSH | r | -0.005 | 0.05 | 0.36 | 0.05 | | 0.08 | -0.20 | 0.03 | 0.14 |
|  | *p* | 0.98 | 0.85 | 0.15 | 0.85 | | 0.77 | 0.42 | 0.92 | 0.56 |
| Estradiol | r | 0.24 | -0.07 | -0.25 | -0.08 | | -0.18 | 0.21 | -0.08 | -0.19 |
|  | *p* | 0.33 | 0.76 | 0.31 | 0.76 | | 0.47 | 0.39 | 0.74 | 0.44 |
| Testosterone | r | -0.42 | -0.08 | -0.34 | -0.08 | | -0.15 | -0.38 | -0.15 | -0.24 |
|  | *p* | 0.07 | 0.73 | 0.17 | 0.73 | | 0.55 | 0.10 | 0.53 | 0.32 |
| Progesterone | r | 0.004 | -0.42 | -0.18 | -0.32 | | -0.32 | 0.12 | -0.31 | -0.40 |
|  | *p* | 0.99 | 0.08 | 0.46 | 0.18 | | 0.19 | 0.63 | 0.20 | 0.09 |
| insulin | r | -0.32 | -0.23 | -0.25 | -0.23 | | 0.02 | -0.09 | -0.23 | -0.34 |
|  | *p* | 0.21 | 0.38 | 0.35 | 0.38 | | 0.94 | 0.72 | 0.37 | 0.18 |

Correlations between hormones and the relative abundance of microbiota are shown, based on Pearson correlation tests. The levels of cortisol were measured three times over 24 h, and the levels of other hormones were measured in the morning (ZT6), including LH, FSH, oestradiol, testosterone, progesterone, and insulin.
